# Temporal Trends of Suicide Mortality in Mainland China: Results from the Age-Period-Cohort Framework

**DOI:** 10.3390/ijerph13080784

**Published:** 2016-08-03

**Authors:** Zhenkun Wang, Jinyao Wang, Junzhe Bao, Xudong Gao, Chuanhua Yu, Huiyun Xiang

**Affiliations:** 1 School of Public Health, Wuhan University, Wuhan 430071, China; wongzhenkun@gmail.com (Z.W.); jinjinyao456@163.com (J.W.); junzhe_bao@126.com (J.B.); gaoxudongne@163.com (X.G.); 2 Center for Injury Research and Policy & Center for Pediatric Trauma Research, The Research Institute at Nationwide Children’s Hospital, Columbus, OH 43210, USA; 3 College of Medicine, The Ohio State University, Columbus, OH 43210, USA; 4 Global Health Institute, Wuhan University, Wuhan 430071, China

**Keywords:** suicide, APC framework, temporal trend, China

## Abstract

The aim of this study is to explore the long-term trends of suicide mortality in China. We implemented the age-period-cohort (APC) framework, using data from the Global Burden of Disease Study 2013. Our results showed that the net drift of suicide mortality was −4.727% (95% CI: −4.821% to −4.634%) per year for men and −6.633% (95% CI: −6.751% to −6.515%) per year for women, and the local drift values were below 0 in all age groups (*p* < 0.01 for all) for both sexes during the period of 1994–2013. Longitudinal age curves indicated that, in the same birth cohort, suicide death risk increased rapidly to peak at the life stage of 20–24 years old and 15–24 years old for men and women, respectively, and then showed a decelerated decline, followed by a rise thereafter after 54 years old for men and a slight one after 69 years old for women. The estimated period and cohort RRs were found to show similar monotonic downward patterns (significantly with *p* < 0.01 for all) for both sexes, with more quickly decreasing for women than for men during the whole period. The decreasing trend of suicide was likely to be related to the economic rapid growth, improvements in health care, enhancement on the level of education, and increasing awareness of suicide among the public in China. In addition, fast urbanization and the effective control of pesticides and rodenticides might be the special reasons behind these trends we observed in this study.

## 1. Introduction

Suicide, a serious public health issue worldwide [1], can be defined as fatal, intentional, self-inflicted injury with the intent to end life [2]. In 2013 alone, according to a recent report [3] from the Global Burden of Disease (GBD) Mortality and Causes of Death Collaborators, about 842,400 suicide deaths occurred worldwide, which caused 34,938,784 years of life lost (YLL). The World Health Organization reported that [4] suicide is a leading cause of death worldwide and is the second-leading cause of death for those among 15–29 years old globally. In China, the world’s most populous country, the situation is also gloomy: suicide is the leading cause of death of 15–34 year olds and ranks as the fifth leading cause of death for the general population. It has been reported that China’s suicide deaths account for one-quarter to one-third of the whole world annual total [5].

Not until 1989 did the Chinese government release suicide data for mainland China. Since then many studies have focused on suicide in China, especially the epidemiological aspects. Existing studies [5,6,7,8,9,10,11,12,13,14,15,16,17,18,19,20,21] showed that the suicide rate in China was very high but it has fallen rapidly and is now below the world average; the suicide rate of Chinese women was higher than that of men but it is now lower; the elderly were the vulnerable population; differences in rates of suicide exist between urban and rural areas. However, how the suicide risk differs across Chinese people’s lifespan; what are the annual percentage changes of suicide rates in various age groups and how are the relative risks of period and cohort and what are the underlying possible reasons behind still remain unknown. To try to answer these questions, we here investigated the long-term trends of suicide mortality between 1994 and 2013 with the aid of age-period-cohort framework using data from the Global Burden of Disease Study 2013 (GBD 2013). The findings of this study could provide a better understanding of suicide epidemiology in China, as well as provide useful guidelines for the prevention and control of suicide deaths.

## 2. Materials and Methods

Data used in this study were obtained from the GBD 2013, a large international cooperation project which provided internally consistent estimates of age-sex specific all-cause and cause-specific mortality for 240 causes of death globally, regionally and nationally from 1990 to 2013 [3]. There were five main data sources [22] that GBD 2013 adopted to estimate data on causes of death in China, and suicide data of this study were mainly extracted from three of them, i.e., Disease Surveillance Points (DSPs), Maternal and Child Surveillance System and Chinese Center for Disease Control and Prevention (CDC) Cause of Death Reporting System. Suicide was identified based on the International Classification of Disease (ICD). It should be noted that during our study period, there was a transition from the 9th to 10th revision of the ICD (Codes E950-959 in ICD-9, and X60-84.9 in ICD-10) [22], but it has been proven that ICD changes had no substantial impact on the analysis of temporal trends of suicide [23]. To describe the mortality trends, the suicide rates for males and females in China were age-standardized by the GBD 2013 global age-standard population [22].

Age, period and cohort (APC) analysis attempts to assess the effects of age, period and cohort on the outcome such as demographic or disease rates. It is a helpful tool broadly adopted in the fields of demography, sociology and epidemiology. The age effects represent a differing risk of the outcome; the period effects represent variations in the outcome over time that influence all age groups simultaneously; the cohort effects are associated with changes of the outcome across groups of individuals with the same birth years [24,25]. Due to the well-known non-identification problem (Age = Period − Cohort), it is hard to estimate the unique set for each age, period and cohort effect in APC model. In fact since the 1970s [26], many researchers have stated that it is “futile” and “impossible” rather than hard to estimate them [27]. This is because the collinearity is inherent in the process that creates the data [28], so although various attempts have emerged in the last four decades to separate one effect from the other two, none of them is really effective and widely accepted. According to Bell and Jones [28,29], these attempts will only work “in very specific and arguably usually unrealistic circumstances”.

Holford chose another way to deal with the problem. His method avoids directly separating contributions of age, period and cohort, and focuses on estimable functions instead which are invariant to the particular constraint applied [30]. He suggested if age, period, and cohort trends are orthogonally decomposed into their linear and nonlinear parts [30], many useful functions can be estimated, including [25,31] net drift, longitudinal age trend, and age, period and cohort deviations. In addition, it has been proven that other useful estimable functions such as local drifts [32], longitudinal age curve [33], the period rate ratio (RR) [34] and the cohort RR [35] can be obtained too.

In this study, we mainly focused on the following estimable functions [36]: net drift, the overall log-linear trend by calendar period and birth cohort, indicating the overall annual percentage change; local drifts, the log-linear trend by calendar period and birth cohort for each age group, indicating annual percentage changes for each age group; longitudinal age curve indicating the fitted longitudinal age-specific rates in reference cohort adjusted for period deviations; the period (or cohort) RR indicating the period (or cohort) relative risk adjusted for age and non-linear cohort (or period) effects in a period (or cohort) versus the reference one. To conduct APC analysis, the mortality and population data were arranged into consecutive 5-year periods from 1994 to 2013 (data from 1990 to 1993 were not considered because they were not enough for a 5-year period) and successive 5-year age intervals from 10–14 years to 75–79 years. Since the occurrence of suicide in those below 10 years old was very rare, and individuals over 80 were only recorded as one group in GBD database, they were not considered in this study. We obtained these estimable parameters by the age-period-cohort Web Tool (Biostatistics Branch, National Cancer Institute, Bethesda, MD, USA). The central age group, period, and birth cohort were defined as the reference respectively in all age-period-cohort analyses; in case of an even number of categories, the reference value was set as the lower of the two central values [36].

## 3. Results

**Figure 1:**
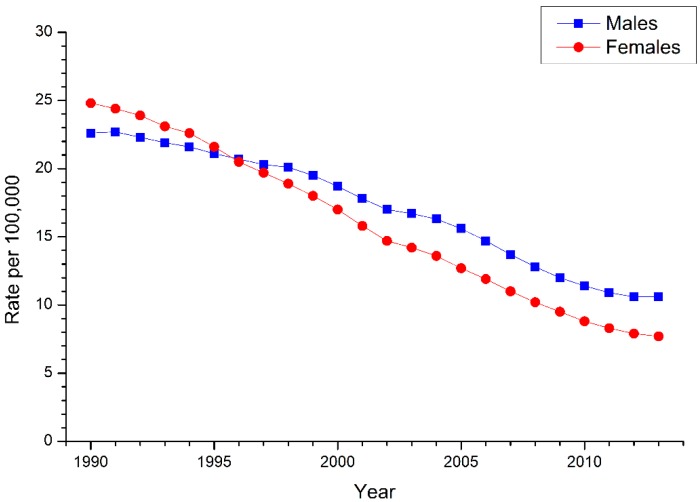
Trends of age-standardized suicide death rates per 100,000 population by gender in China, 1990–2013.

The trends of age-standardized mortality rate (ASMR) of suicide by gender in China from 1990 to 2013 are shown in Figure 1. During this period, we can observe that the ASMRs of suicide of Chinese males and females showed continuous declining trends, from 22.6 to 10.6 per 100,000 for men and 24.8 to 7.7 per 100,000 for women. Our results indicated that the ASMR of suicide of women had declined faster than that of men. The reduction in ASMR of suicide from 1990 to 2013 was over a half in both genders (53.1% for men and 69.0% for women). It should be noted that since 1996, the ASMR of suicide of females has been lower than that of males.

**Figure 2:**
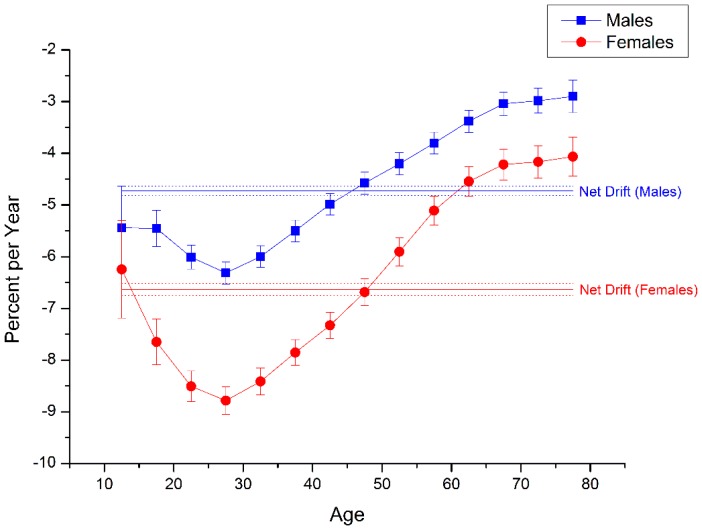
Local drift with net drift values for suicide rates by gender in China. Age group specific annual percent change (%) in the mortality rates of suicide and the corresponding 95% confidence intervals by gender in China.

The net drift and local drift values, which represented annual percentage change of the expected age-adjusted rates and of the expected age-specific rates over time respectively, were displayed in Figure 2. During the period of 1994–2013, the overall net drift was −4.727% (95% CI: −4.821% to −4.634%) per year for men and −6.633% (95% CI: −6.751% to −6.515%) per year for women (*p* < 0.01 for both), which could be both regarded as substantial ( their values were not in the range of −1% to 1% per year) [18]. We observed that local drift values were below 0 in all age groups in both sexes and in each age group. The curves of local drifts for both sexes first decreased with age with a negative peak at the age group of 25–29, followed by a monotonic increase thereafter. Our results indicated that all local drift values of females were below their counterparts of males. The difference between the local drift values of females and males became larger from age group 10–14 to 25–29 and then began to become smaller.

**Figure 3:**
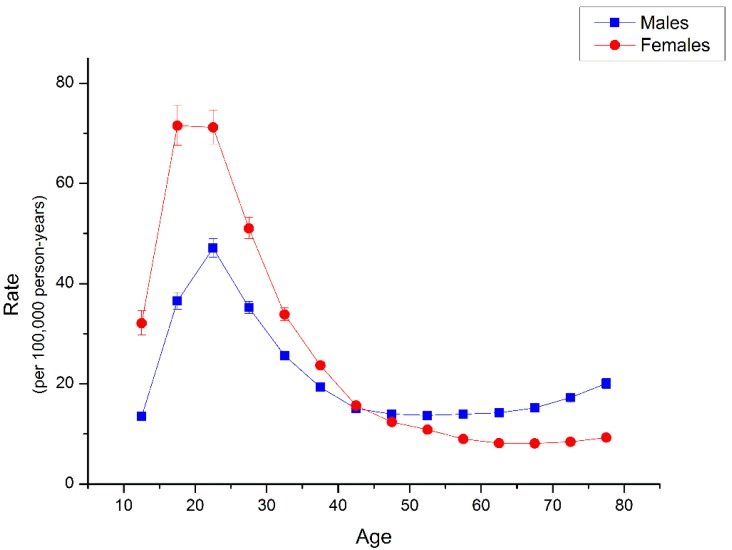
Longitudinal age curves of suicide rate by gender in China. Longitudinal age curves of the suicide rates (per 100,000 person-years) and the corresponding 95% confidence intervals (some of them were too narrow to show in the figure) by gender in China.

The longitudinal age curves of suicide rate by gender were illustrated in Figure 3. In the same birth cohort, suicide risk increased rapidly to peak at the life stage of 20–24 years old and 15–24 years old for men and women respectively, and then showed a decelerated decline, followed by a rise thereafter after 54 years old for men and a slight one after 69 years old for women. Suicide risk was higher in females than in males between age group 10–14 and 40–44, but it began to be lower in females than in males from the age group 45–49. The difference between suicide risk of females and males became larger from age group 10–14 to 15–19 and then began to become smaller. There was nearly no difference between suicide risks of females and males in age group 40–44, and the difference became reversed from age group 45–49 and then began to become larger.

**Figure 4:**
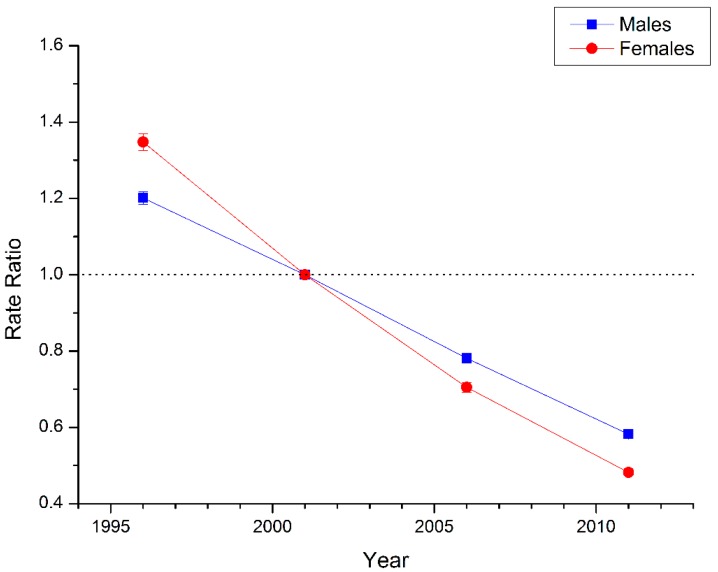
Period RRs of suicide rate by gender in China. Period effects obtained from age-period-cohort analyses for the suicide rates and the corresponding 95% confidence intervals (some of them were too narrow to show in the figure) by gender in China.

**Figure 5:**
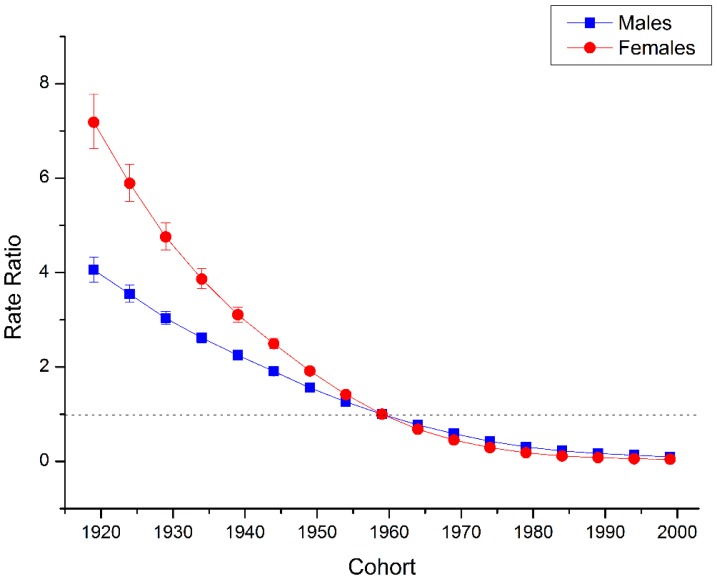
Cohort RRs of suicide rate by gender in China. Cohort effects obtained from age-period-cohort analyses for the suicide rates and the corresponding 95% confidence intervals (some of them were too narrow to show in the figure) by gender in China.

The estimated period and cohort RRs by gender were displayed in Figure 4 and Figure 5 respectively. The period RRs were found to have similar monotonic decreasing patterns for both sexes, with more quickly declining for women than for men during the whole study period. Similarly, the cohort RRs were also found to show similar monotonic downward patterns for both sexes, with more quickly declining for women than for men in all the successive cohorts.

The declining trends of both sexes witnessed a decelerated process, and tended to be smoother from cohort around 1970. In addition, based on the specific results of Wald tests (see Table 1), there were statistically significant cohort and period RRs for both sexes (*p* < 0.01 for all), and so were the net drift and local drifts (*p* < 0.01 for all).

## 4. Discussion

This is the first published study, to our knowledge, to explore the long-term trends of suicide mortality in China between 1994 and 2013 with the aid of age-period-cohort framework. Like the existing reports, the results of age-standardized death rates for suicide showed that suicide of both genders showed continuous declining trends, with suicide rate of females declining faster. The suicide ASMR of Chinese females was higher than that of males but is now lower. Our local drift findings further indicated that suicide mortality in all the age groups (10–79 years old) showed an estimated annual percentage decrease over the study period, with the biggest decrease in age group 25–29 years old for both genders. The estimated annual percentage decrease was greater for females than males in all the age groups, and it should be noted that this local drift gap between females and males was larger among the groups of the young and middle-aged (15–49 years old) than other age groups.

Age is one of the most important demographic factors for suicide. This is because suicide risk varies from different life stages due to physiological changes, social part or status changes, life stress, or a blend of these [24]. Longitudinal age curve results (see Figure 4) showed that, in the same birth cohort, Chinese people’s life stage at the greatest suicide risk was the 20–24 years old for men and 15–19 years old for women, and suicide risk showed a slight increase after their 60 years old. However, previous studies found that although age effect curves varied in APC analyses from many developed regions, including North American countries (such as in the U.S. [37]), European regions (such as in Swiss [38], Sweden [39], England and Wales [40]), and Asian areas (in such as Japan [41], South Korea [42] and Hong Kong [43]), the results of these studies all showed that the greatest risky life stage for suicide was after 50 years old. The only studies whose results were somehow similar to ours were the APC analyses of the U.S. black population [44,45], which also showed the riskiest life stage for suicide was the youth. Our study suggested that the special age pattern of suicide in China merits attention and the underlying reasons warrant further research.

Nevertheless, based on these existing studies [13,14,46,47], we can speculate that high hopelessness, interpersonal disputes, life pressures, lower socioeconomic status/quality of life, lower education/higher school dropout, and less likely to seek help or report for depressive symptoms, suicidal ideation, and suicide attempts might be contributing factors for young adults at the greatest suicide risk in China. The plausible reasons why suicide risk slightly increased in old life stage of Chinese population might be retirement, “empty-nest crisis”, death of relatives (especially spouse) and/or friends, physical limitations to mobility, and serious illness that all likely lead to more severe isolation in later life [37,48], just like the case in those developed regions, given the fact that empirical studies have confirmed a connection between social isolation and inclination toward suicide among old people [48]. Our longitudinal age curves indicated that in the same birth cohort, suicide death risk was greater in Chinese females than males, but this difference almost disappeared in the 40–44 years old groups and became reversed thereafter. This finding provided useful evidence for suicide prevention in China. For instance, for students or other young people groups, more attention should be paid to the prevention and control of female suicide; on the other hand, suicide prevention efforts should focus on male suicide in old life stage in China.

Although the period effect and cohort effect could be estimated separately as the period RR and cohort RR by certain restriction (adjusting for deviation [36] in this study), it is often not easy to interpret their results separately in the real world. This is because in many cases, the period effect often influences certain age group(s) more or less when influences all age groups simultaneously, which leads to some cohort effect. Cohort effect essentially reflects different birth cohorts’ various risk, but in reality, different cohorts were born in different periods and thus inevitably have confounding impact on period effect to some extent. So we here tried to systematically discuss the possible reasons for the decreasing trends of period and cohort effects.

The most possible factor that could influence suicide rates is the economic conditions [49,50]. It is generally known that China’s economy has experienced a spectacular growth since the 1980s. Higher income per capita and better living standard could eliminate lots of possible conflicts related to poverty and familial relationships, which have been regarded as important causes [51] for suicide in China, especially for the people in rural areas. In addition, improvements in health care—both in treatment and accessibility for mental and substance abuse disorders, more school education for all, and increasing awareness of suicide among the public might be the explanations for the decreasing suicide trend in China. For example, better education could improve abilities in problem tackling, conflict resolution, and skills for managing disputes, which all are regarded as protective factors for suicide. More knowledge on suicide could improve help-seeking from the family, friends or specialists, and these helps could effectively reduce suicide risk.

Urbanization [44] is a relatively special and important contributing factor for China’s suicide mortality trend. According to the National Bureau of Statistics of China, the proportion of residents living in urban areas had a dramatic increase from 26% in 1990 to 50% in 2010 and 56% in 2015 [52]. Fast urbanization, on one hand, could increase the rescue success rate of suicides since more people now have better access to medical and emergency services. These services are usually centered in urban areas, and in the past, lots of suicide deaths in China could be attributed to unsuccessful or delayed medical resuscitation. On the other hand, fast urbanization might have contributed to a reduced availability of highly toxic pesticide products in households because pesticides were mainly used in farming activities in rural communities [51]. Meanwhile, most lethal pesticides and rodenticides have been banned by law for agriculture or other use in recent decades in China [53]. Both of these developments were considered by researchers to play an important role in the decreasing suicide trend, because consumption of lethal pesticides or rodenticides is the most popular method for suicide in China (accounting for more than half of suicide deaths) [15,54,55] due to a great extent to their availability [56].

Several limitations in the present study should be mentioned. First, although many steps were taken, including steps to address incompleteness, under-reporting, and misclassification corrections [57] as well as the redistribution of garbage codes (undetermined injury death, ICD-9 codes E980–989 and ICD-10 codes Y10–Y34 andY872) [58,59] in the GBD 2013 to enhance the data quality and comparability, data in this study were likely to be still influenced by the quality issue to certain extent. So it was possible that suicide death data quality might bias our findings. But we believed that the bias has been well controlled after those correction and adjustment steps. This issue has been studied in Salmerón et al. and the authors showed that [60] the trend obtained using only deaths catalogued as suicide, and the trend obtained using deaths catalogued as suicide plus deaths of undetermined intent, are very similar. Second, like other APC analyses, there was the inevitability to be affected by ecological fallacy to some extent in this study, which means interpretations of results at population level do not necessarily hold for individuals. Therefore, all findings from the APC analysis in this study still need further confirmation in future individual-based studies.

## 5. Conclusions

In summary, our study showed that the net drift of suicide mortality was −4.727% (95% CI: −4.821% to −4.634%) per year for men and –6.633% (95% CI: −6.751% to −6.515%) per year for women, and the local drift values were below 0 in all age groups (*p* < 0.01 for all) in both sexes during the period of 1994–2013 in China. Longitudinal age curves indicated that, in the same birth cohort, suicide death risk increased rapidly to peak at the 20–24 years old life stage and 15–24 years old for men and women, respectively, and then showed a decelerated decline followed by a rise thereafter after 54 years old for men and a slight one after 69 years of age for women. The estimated period and cohort RRs were found to show similar monotonic downward patterns (significantly with *p* < 0.01 for all) for both sexes, with more quickly decreasing for women than for men during the whole period. The decreasing trend of suicide was likely to be caused by the economic rapid growth, improvements in health care, more school education, and increasing awareness of suicide among the public. In addition, fast urbanization and the effective control of pesticides and rodenticides might be the special reasons behind the declining suicide trend for both sexes during the past two decades in China.

## Figures and Tables

**Table 1:** Wald Chi-Square tests for estimable functions in the APC model.

Null Hypothesis	Males		Females	
	Chi-Square	*p*-Value	Chi-Square	*p*-Value
Net Drift = 0	9386.6	<0.01	11,294	<0.01
All Period RR = 1	9390.7	<0.01	11,513	<0.01
All Cohort RR = 1	10,298	<0.01	12,444	<0.01
All Local Drifts = Net Drift	721.41	<0.01	930.6	<0.01

## Abbreviations

The following abbreviations are used in this manuscript:

GBD 2013	The Global Burden of Disease Study 2013
ASMR	age-standardized mortality rate
APC analysis	age-period-cohort analysis
